# Quantitative Proteomic Analysis of Four Developmental Stages of *Saprolegnia parasitica*

**DOI:** 10.3389/fmicb.2017.02658

**Published:** 2018-01-11

**Authors:** Vaibhav Srivastava, Svetlana Rezinciuc, Vincent Bulone

**Affiliations:** ^1^Division of Glycoscience, Royal Institute of Technology, AlbaNova University Centre, Stockholm, Sweden; ^2^ARC Centre of Excellence in Plant Cell Walls and School of Agriculture, Food and Wine, The University of Adelaide, Adelaide, SA, Australia

**Keywords:** *Saprolegnia*, cysts, mycelium, quantitative proteomics, pathogen, fish

## Abstract

Several water mold species from the *Saprolegnia* genus infect fish, amphibians, and crustaceans in natural ecosystems and aquaculture farms. *Saprolegnia parasitica* is one of the most severe fish pathogens. It is responsible for millions of dollars of losses to the aquaculture industry worldwide. Here, we have performed a proteomic analysis, using gel-based and solution (iTRAQ) approaches, of four defined developmental stages of *S. parasitica* grown *in vitro*, i.e., the mycelium, primary cysts, secondary cysts and germinated cysts, to gain greater insight into the types of proteins linked to the different stages. A relatively high number of kinases as well as virulence proteins, including the ricin B lectin, disintegrins, and proteases were identified in the *S. parasitica* proteome. Many proteins associated with various biological processes were significantly enriched in different life cycle stages of *S. parasitica*. Compared to the mycelium, most of the proteins in the different cyst stages showed similar enrichment patterns and were mainly related to energy metabolism, signal transduction, protein synthesis, and post-translational modifications. The proteins most enriched in the mycelium compared to the cyst stages were associated with amino acid metabolism, carbohydrate metabolism, and mitochondrial energy production. The data presented expand our knowledge of metabolic pathways specifically linked to each developmental stage of this pathogen.

## Introduction

Oomycetes are filamentous eukaryotic microbial organisms (Phillips et al., 2008; Bruno et al., 2009). Many oomycetes are pathogenic to plants or animals, and cause economic and environmental losses in natural and agricultural environments (Phillips et al., 2008; Jiang and Tyler, 2012). One of the best studied oomycetes is the plant pathogen *Phytophthora infestans*, the causal agent of late blight of potato (Tyler et al., 2006; Jiang and Tyler, 2012), but less is known about species that belong to other genera. Saprolegniales species include pathogens of amphibians, crustaceans, fish, and insects (van West, 2006; Bruno et al., 2009). Species from this order are also known to infect fish eggs, resulting in cell death (Robertson et al., 2009). Members of the *Saprolegnia* genus are likely present in all fresh water ecosystems, and may be partially responsible for the global decline in wild fish stocks and amphibian populations (Kiesecker et al., 2001; Pounds, 2001; Neitzel et al., 2004). The species *Saprolegnia parasitica* causes Saprolegniasis, a disease characterized by visible white or gray patches of filamentous mycelium on the body or fins of freshwater fish (van West, 2006).

The life cycle of *S. parasitica* includes clearly defined developmental stages and reproduction occurs both sexually and asexually (Phillips et al., 2008). Sexual reproduction starts with the formation of antheridia and oogonia, which fuse together to produce oospores (Phillips et al., 2008). Under starvation conditions, asexual reproduction dominates: the hyphal cells of the mycelium form sporangia at their tip, which release primary zoospores. Following the encystment of primary zoospores to form primary cysts, secondary zoospores are released. Secondary zoospores subsequently encyst, leading to the formation of secondary cysts, which are characterized by the presence of long hooked hairs that presumably assist in attachment to a suitable host (Söderhäll et al., 1991; van West, 2006). Secondary cysts also have the ability to release zoospores. When secondary zoospores find a suitable host, they germinate and differentiate into hyphal cells and eventually mycelium in the host tissue, which initiates the infection process.

To date, plant pathogenic oomycetes are better studied than their animal counterparts, although both have substantial economic impact on the industries they affect (van West, 2006; Phillips et al., 2008; Bruno et al., 2009). In a study comparing animal and plant pathogenic oomycetes, it was found that *Saprolegnia* lacks families of effector proteins such as RXLR, CHXC, and Crinkler proteins (Jiang et al., 2013), which allow plant pathogenic oomycetes to enter their host cells (Haas et al., 2009). It was reported that some pathogenesis-related genes in *Saprolegnia* have been acquired from the host or other animal pathogens via horizontal gene transfer (HGT) (Jiang et al., 2013). In addition, the *S. parasitica* genome contains many unique protein family domains that are absent in other oomycetes as well as a large number of genes that are not orthologous to any known genes in other species (Jiang et al., 2013). Combined, this information suggests the proteins involved in the infection processes of plant and animal pathogenic oomycetes differ greatly.

In recent years, the global increased aquaculture production combined with the banning of chemicals used to control *Saprolegnia* infections, including the carcinogenic compound malachite green, have led to the spread of *Saprolegnia* populations worldwide (van West, 2006). Although other chemicals are being trialed for their potential to control *Saprolegnia* species (Pottinger and Day, 1999; Gieseker et al., 2006; Rezinciuc et al., 2014; Warrilow et al., 2014), there are currently no effective and safe chemical treatments that give sufficient protection against Saprolegniasis. Therefore, alternative measures for disease control are urgently needed in order to manage the spread of the infection. Little is known about the biology and infection processes of *Saprolegnia* at the molecular and cellular levels and it is only rather recently that the *S. parasitica* genome has been sequenced (Jiang et al., 2013). One path with potential for the development of novel methods of disease control is the identification and targeting of specific proteins involved in the establishment of infection across developmental stages of *S. parasitica.* Previous proteomic studies on oomycetes have essentially focused on plant pathogens, and were based on both global and targeted mass spectrometry. For example, differentially regulated proteins were identified from the germinating cysts and appressoria of *P. infestans*, using two-dimensional gel electrophoresis (2-DE) and mass spectrometry (Ebstrup et al., 2005) whereas Meijer et al. (2014) profiled the secretome and extracellular proteome of *P. infestans*. Several comparative proteomic analyses have also been performed on other *Phytophthora* species (Savidor et al., 2008; Hosseini et al., 2015; Pang et al., 2017).

Here, we present a comparative proteomic analysis examining four *S. parasitica* developmental stages, namely the mycelium, primary cysts, secondary cysts, and germinated cysts. Functional classification of all enriched proteins revealed their involvement in different biological processes associated with each developmental stage. Candidate proteins potentially involved in both vegetative growth and infection processes were identified. To date, this is the most comprehensive study using quantitative proteomics to examine a *Saprolegnia* proteome. The variations uncovered between different *S. parasitica* developmental stages promise to enhance our current knowledge of the biology of the pathogen and can potentially be exploited in disease management strategies by targeting proteins specifically expressed at key infectious stages.

## Materials and Methods

### Preparation of *Saprolegnia parasitica* Life Cycle Stages

**FIGURE 1:**
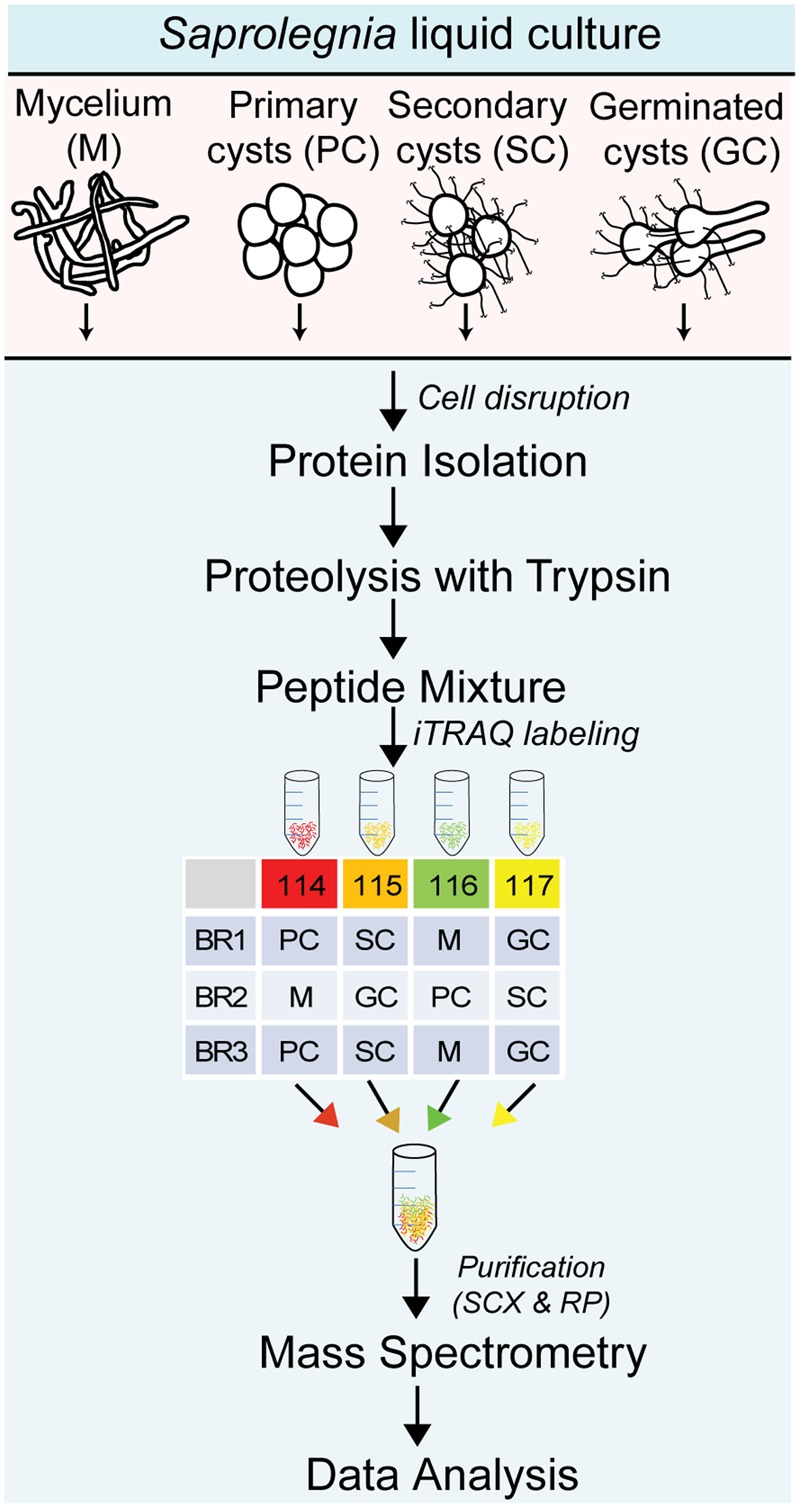
Experimental workflow used for the quantitative proteomic analysis of different life cycle stages of *Saprolegnia parasitica.* M, mycelium; PC, primary cysts; SC, secondary cysts; GC, germinated cysts; iTRAQ, isobaric tags for relative and absolute quantitation; BR1, biological replicate 1; BR2, biological replicate 2; BR3, biological replicate 3; SCX, strong cation-exchange chromatography; RP, reversed-phase chromatography.

Cultures of *S. parasitica* cells were grown in a PC2 laboratory. The living cells remaining after protein preparation from the different developmental stages were autoclaved. Four developmental stages of *S. parasitica* (CBS223.65), including mycelium (M), primary cysts (PC), secondary cysts (SC), and germinated cysts (GC) were isolated and examined in this study. Three independent biological replicates for each developmental stage were prepared from independent mycelial cultures (**Figure 1**). *Saprolegnia* cultures were maintained on peptone-glucose agar medium (Unestam, 1965). The protocol described by Diéguez-Uribeondo et al. (1994) was used to induce sporulation. The mycelium was first grown for 2 days at room temperature (RT) in a peptone-glucose liquid medium. Next, the mycelium was washed three times, followed by an overnight incubation in filtered and autoclaved lake water, a step that induces sporulation and the release of zoospores. The mycelium was inspected regularly for the formation of primary and secondary zoospores, which were then collected separately. For both primary and secondary cyst production, the zoospores were encysted by vigorous agitation for 1 min using a vortex operated at 14,000 rpm. The primary and secondary cysts were collected by centrifugation at 5000 × *g* for 5 min. The secondary cysts were subsequently incubated for 12 h at RT to form germ tubes and the resulting germinated cysts were collected for analysis. The purity of the different cyst stages was also verified by optical microscopy based on the presence of long hairs in SC only (Söderhäll et al., 1991). Cells collected at all stages were frozen immediately after collection in liquid nitrogen and stored at -80°C until protein and RNA extraction.

### Protein Extraction and Sample Preparation for Qualitative and Quantitative Proteomics

The frozen samples were freeze dried, weighed (∼10 mg) and disrupted using a MM400 Mixer Mill (Retsch, Germany) for 10 min at 30 beats/sec. The resulting powder was resuspended in extraction buffer [3% sodium deoxycholate (SDC) in 50 mM triethylammonium bicarbonate (TEAB) pH 8.0, 1 mM ethylenediaminetetraacetic acid (EDTA) and protease inhibitor (cOmplete, Mini, EDTA-free, Roche)] and the total protein concentration was determined using the Bradford assay.

For quantitative proteomics, aliquots of mycelial (M) and cyst (PC, SC, and GC) samples containing 100 μg proteins each were prepared as described above and diluted three times with 50 mM TEAB to reach a final SDC concentration of 1%. Protein disulfide bonds were reduced for 1 h at 60°C in the presence of 5 mM *tris*-(2-carboxyethyl)-phosphine, and the resulting free thiol groups were alkylated at RT for 15 min in the presence of 10 mM methyl methanethiosulfonate. Trypsin (Promega, Madison, WI, United States) was added to each sample at an enzyme-to-protein ratio of 1:10 and the mixtures were incubated at 37°C for 16 h. The solutions were acidified by the addition of trifluoroacetic acid (TFA) to a final concentration of 0.5% and centrifuged to remove the sodium deoxycholate. The resulting supernatants were transferred to new tubes and dried under vacuum. The dried peptides were dissolved in 100 μl of a mixture consisting of 25% 250 mM triethylammonium bicarbonate and 75% (v/v) ethanol, and subsequently labeled with iTRAQ reagents (isobaric tags for relative and absolute quantitation, 114-117; AB SCIEX, Foster City, CA, United States) according to the manufacturer’s instructions. The iTRAQ tags used for the biological replicates are shown in **Figure 1**. The labeled peptides from each biological replicate were combined (**Figure 1**), dried and re-suspended in 10 mM ammonium formate pH 3.0 containing 10% acetonitrile (loading buffer). The mixtures were then loaded onto 1-ml NuviaTM HR-S cartridges (Bio-Rad, Germany) that were prepared according to the manufacturer’s instructions using a peristaltic pump. After washing the cartridges with loading buffer, the peptides were eluted at a rate of 0.2 ml/min by the sequential addition of ammonium formate salt plugs of 1.5 ml each (50, 75, 100, 125, 150, 175, 200, 225, 250, 275, 300, 325, 350, and 400 mM in 20% acetonitrile; pH 3.0). Each fraction was dried and desalted using C18 Spin Columns (Thermo Scientific, United States) for mass spectrometric analysis.

For SDS-PAGE analysis of all developmental stages, 1 mg of each powdered sample was boiled in SDS buffer [75 mM Tris-HCl buffer pH 6.8 containing 3% (w/v) SDS, 100 mM DTT, 15% (w/v) glycerol and 0.002% bromophenol blue] for 5 min at 95°C. The resulting mixture was centrifuged to remove any insoluble material and the supernatant was loaded on a 10% mini-Protean TGX SDS-PAGE system (Bio-Rad). After staining with Coomassie Blue (ThermoScientific, United States), each lane of the gel was cut into 30 bands of similar volume (Supplementary Figure S2) from the top to the bottom of the gel and the proteins were subjected to in-gel digestion with trypsin and the resulting peptides were analyzed by mass spectrometry as previously described (Srivastava et al., 2013).

### Nano-LC-MS/MS Analysis of Samples Subjected to iTRAQ Labeling

Reverse-phase LC-electrospray ionization-MS/MS analysis of the peptide samples subjected to iTRAQ labeling was performed using a nanoACQUITY ultra performance liquid chromatography (UPLC) system (Waters, Milford, MA, United States) coupled to a Q-TOF mass spectrometer (Xevo Q-TOF, Waters, Milford, MA, United States). The purified peptide fractions corresponding to each salt plug were resuspended in 0.1% TFA, loaded onto a C18 trap column (Symmetry 180 μm × 20 mm, 5 μm; Waters, Milford, MA, United States) and washed with 0.1% (v/v) formic acid at a rate of 15 μl/min for 10 min. The samples eluted from the trap column were then separated on a C18 analytical column (75 μm × 200 mm, 1.7 μm; Waters, Milford, MA, United States) at a rate of 225 nl/min using 0.1% formic acid as solvent A and 0.1% formic acid in acetonitrile as solvent B. The proportion of solvent B varied as follows: 0.1–8% B (0–5 min), 8–25% B (5–185 min), 25–45% B (185–201 min), 45–90% B (201–205 min), 90% B (205–213 min), and 90–0.1% B (213–215 min). The eluting peptides were sprayed into the mass spectrometer with the capillary and cone voltages set to 2.3 kV and 45 V, respectively. The five most abundant signals from a survey scan (400–1300 m/z range, 1 s scan time) were then selected by charge state, and the appropriate collision energy was applied for sequential MS/MS fragmentation (50–1800 m/z range, 1 s scan time).

### Data Processing, Protein Identification, and Quantification

Our in-house Automated Proteomics Pipeline, which automates the processing of proteomics tasks such as peptide identification, validation, and quantitation from LC–MS/MS data and allows easy integration of many separate proteomic tools (Malm et al., 2014), was used to analyze the MS data. The raw MS data file was first analyzed using the Mascot Distiller software (version 2.4.3.2, Matrix Science, London, United Kingdom) and the resulting mgf files were converted into the mzML file format using msconvert (Kessner et al., 2008). The *Saprolegnia* protein database (20,088 entries) was then searched using several search engines in parallel, i.e., MS-GF+ (Kim et al., 2010) v1.0 (v8299), MyriMatch (Tabb et al., 2007) (version 2.1.120), Comet (Eng et al., 2013) (version 2013.01 rev.0) and X!Tandem (Craig and Beavis, 2004) (version 2011.12.01.1; LabKey, Insilicos, ISB, Seattle, WA, United States). The following settings were used for the searches: trypsin specific digestion with two missed cleavages allowed; peptide tolerance of 200 ppm; fragment tolerance of 0.5 Da; methylthio on Cys and iTRAQ 4-plex for peptide N-t and Lys used as fixed modifications; oxidized Met and Tyr for iTRAQ 4-plex analysis in variable mode. The results from all search engines were validated by PeptideProphet (Keller et al., 2002). Protein quantitation was performed from the intensities of the iTRAQ reporter ions, which were extracted using the TPP tool Libra (Li et al., 2003) (TPP v4.6 OCCUPY rev 3) after the isotopic correction factors provided by the manufacturer of the iTRAQ reagent had been applied. The iTRAQ channels were normalized using the sum of all the reporter ion intensities from each iTRAQ channel and equalizing each channel’s contribution by dividing the individual reporter ion intensities by the corresponding channel-specific correction factor. The pep.xml files obtained from PeptideProphet were combined using iProphet (Shteynberg et al., 2011) and the protein lists were assembled using ProteinProphet (Nesvizhskii et al., 2003). The final protein ratios were calculated using LibraProteinRatioParser (Li et al., 2003) and a concatenated target-decoy database-search strategy was used to check the false positives rate (<1%) for all searches.

Sequences with a peptide probability cutoff of 0.95 were exported for each protein. Peptides matching two or more proteins (shared peptides) were excluded from the analysis along with proteins that had no unique peptides (i.e., identified by shared peptides only). Proteins identified by one unique peptide were considered as identified whereas those identified by two or more unique peptides were used for the quantitative analysis.

The differences between iTRAQ samples were evaluated by a one-way ANOVA using the statistical computing program R v.3.2.3 (R Core Team, 2014). ANOVA was performed on proteins which were observed in at least two biological replicates and in at least half of the replicates in four sample types (Chavez et al., 2011). Any missing value for a given protein from one of the three biological replicates was replaced by the average value for that protein from the other two corresponding replicates. Briefly, the iTRAQ intensities obtained for each protein were log2 transformed to obtain a normal distribution (Ross et al., 2004). The values were then normalized to the median log values, followed by an ANOVA test as described above.

Normalized iTRAQ intensities with a *p*-value equal to or less than 0.01 were used for principal component analysis (PCA) analysis (R Core Team, 2014), which was carried out with the R function “prcomp,” and plotted using the package gplots v.2.17.0 (Warnes et al., 2015). The mycelium to cyst ratio (M/PC, M/SC, and M/GC) of each protein was also calculated for each of the three biological replicates (Supplementary Table S1). For quantitative changes of proteins, a 1.25-fold cutoff was set to identify the significantly enriched proteins present in at least two biological replicates, among those with a *p*-value < 0.01 from the aforementioned ANOVA analysis (Chavez et al., 2011). In addition, a chi-square test was also performed to validate the differences observed in the number of mycelium- or cyst-enriched proteins associated with the different biological processes. Significant differences (*p*-values < 0.05) are marked with an asterisks in **Figure 3C**. The mass spectrometry proteomics data have been deposited to the ProteomeXchange Consortium via the PRIDE (Vizcaíno et al., 2016) partner repository with the dataset identifier PXD004695.

### Bioinformatic Analysis

The sequences of all identified proteins were analyzed for functional/pathway annotation, number of transmembrane domains, presence of signal peptides and conserved domains. The functional annotation of the identified proteins was performed using the WebMGA tool (Wu et al., 2011)^1^. In order to reconstruct KEGG pathways for all significantly enriched proteins, a web-based server KAAS (KEGG Automatic Annotation Server^2^) was used. KAAS (Moriya et al., 2007) provides functional annotation of genes by BLAST or GHOST comparisons against the manually curated KEGG genes database, resulting in KEGG Orthology assignments and KEGG pathways. The presence of signal peptides was predicted using SignalP (version 4.1) (Petersen et al., 2011) and transmembrane domains were predicted using HMMTOP 2.0 (Tusnady and Simon, 2001). The conserved domains in protein sequences were searched using NCBI’s conserved domain database (Marchler-Bauer et al., 2015)^3^. The MultiExperiment Viewer (MeV version 4.9.0) (Saeed et al., 2006) was used to construct a relative protein abundance heat map.

### RNA Extraction and Quantitative Real-time PCR Analysis

To evaluate how well gene expression levels match our quantitative proteomics data, we performed a quantitative real-time PCR (qPCR) analysis on 20 selected genes. A panel of genes related to carbohydrate metabolism, signal transduction, and energy metabolism were used as representatives of proteins that were most abundant in the mycelium (10 genes) or the cysts (10 genes). Total RNA was extracted from frozen *S. parasitica* mycelium (M) and cyst cells (PC, SC, and GC) using an RNeasy^®^ kit (Qiagen, Hilden, Sweden) according to the manufacturer’s instructions. DNA was removed from the RNA samples using a TURBO DNA-freeTM kit (Ambion, United States) and first strand cDNA synthesis was performed from 1 μg total RNA using the Maxima First Strand cDNA Synthesis Kit (Thermo Fisher Scientific). All primers used in our analyses are listed in Supplementary Table S1. Real-time PCR analyses were performed using a CFX96 real-time PCR detection system (Bio-Rad). The reactions were performed using 5 μl of 2X iQ SYBR Green Supermix (Bio-Rad), 0.5 μM of each primer, 3 μl of 17-fold diluted cDNA (2.7 ng/μl), and nuclease-free water to a final volume of 10 μl in two technical replicates for each of three independent biological experiments. The PCR was performed using the following program: 95°C for 3 min, followed by 40 cycles of 10 s at 95°C, 10 s at 60°C, and 10 s at 72°C. Melting curves were generated at the end of the experiment to check the specificity of the PCR products. The raw data were analyzed using the CFX manager^TM^ software (version 3.0; Bio-Rad) which includes the algorithms to perform relative gene expression with normalization to multiple reference genes over multiple plates. Relative expression levels were calculated by normalizing the data to the geometric mean of two reference genes (tubulin and elongation factor), which were selected from an expression stability analysis of three reference genes (CFX manager^TM^, version 3.0; Bio-Rad). The PCR efficiency for each gene was calculated using Real-time PCR Miner (Zhao and Fernald, 2005), and found to range from 85 to 99%.

## Results

### Identification of the Proteins from Four Developmental Stages of *S. parasitica*

**FIGURE 2:**
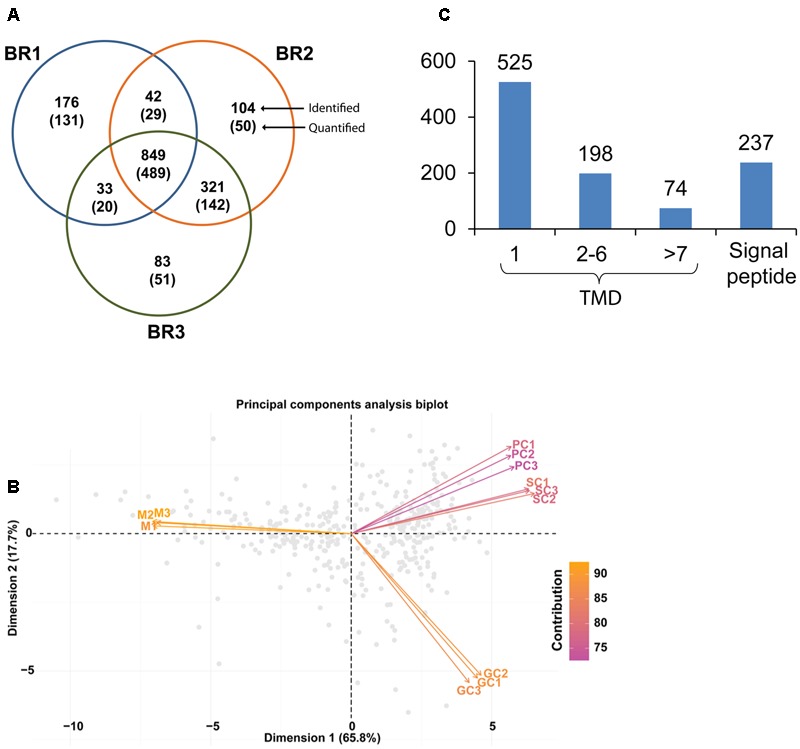
**(A)** Venn diagram showing the number of unique proteins identified and quantified from the iTRAQ analysis of three biological replicates (BR1, BR2, and BR3) corresponding to the four developmental stages; **(B)** principal component analysis (PCA) was performed on all normalized iTRAQ intensities from proteins which had a *p*-value equal to or less than 0.01. The resulting biplot used the two principal component model (PC1 and PC2) with component contributions of 65 and 17%, respectively. The biplot shows proteome data (intensities) as labeled dots for all biological replicates (M1, M2, and M3; PC1, PC2, and PC3; SC1, SC2, and SC3; GC1, GC2, and GC3), with average intensities shown as vectors. Vectors that are closer together are more highly correlated in terms of the observed proteome while vectors that are orthogonal are less correlated; **(C)** total number of identified proteins containing one or more transmembrane domains (TM), and with signal peptides.

In an effort to identify and categorize the proteins that are specific for different developmental stages of *S. parasitica*, we compared the proteome of the mycelium, primary cysts, secondary cysts, and germinated cysts. The purity of the PC and SC was verified by optical microscopy based on the presence of long hooked hairs in SC compared to PC (Söderhäll et al., 1991). A total of 2423 unique proteins were identified across all four developmental stages by combining the data from the gel-based qualitative (1801 unique proteins) and iTRAQ-based quantitative approaches (Supplementary Table S1). The iTRAQ experiments (**Figure 1**) allowed the identification and quantification of 1100 (669 quantified), 1316 (710), and 1286 (702) unique proteins from the three biological replicates BR1, BR2, and BR3, respectively (**Figure 2A** and Supplementary Table S1). Of these, 849 (489) proteins were common to all three biological replicates. Each replicate also contained proteins not identified (or quantified) in the other two replicates, i.e., 176 (131), 104 (50), and 83 (51) proteins were detected exclusively in BR1, BR2, and BR3, respectively (**Figure 2A**). The PCA biplot shows that the mycelium samples were distinctly separate from the primary (PC), secondary (SC), and germinated (GC) cyst samples. The PC, SC, and GC samples were quite similar, although the PC and SC samples were more similar to each other than to the GC samples (**Figure 2B**). In addition, the data revealed that there is little variation between the replicates from each developmental stage (**Figure 2B**). Bioinformatic analysis of all 2423 proteins identified revealed that around 22% (525 proteins) contained one putative transmembrane domain (TMD) and ∼11% (272 proteins) contained two or more TMDs (**Figure 2C**). Furthermore, about 10% (237 proteins) of the proteins analyzed were predicted to contain a signal peptide (**Figure 2C**). The KOG (EuKaryotic Orthologous Groups) database was used for functional classification of all identified proteins^4^. Proteins were grouped into 25 categories according to their putative functional classes as summarized in Supplementary Figure S1. Approximately, 19% (453 proteins) of all identified proteins could not be assigned to any functional category (unclassified) whereas about 9% of the proteins were classified into 2–3 categories (multiple classes) (Supplementary Figure S1). The largest categories correspond to proteins involved in post-translational modifications, protein turnover and chaperones (11%), and translation, ribosomal structure, and biogenesis (9%) (Supplementary Figure S1). Other categories represented include protein involved in energy production (6%), amino acid metabolism (5%), lipid metabolism (3%), carbohydrate transport and metabolism (3%), and signal transduction (4%) (Supplementary Figure S1).

### Differential Abundance of Protein Classes across the *S. parasitica* Developmental Stages

**FIGURE 3:**
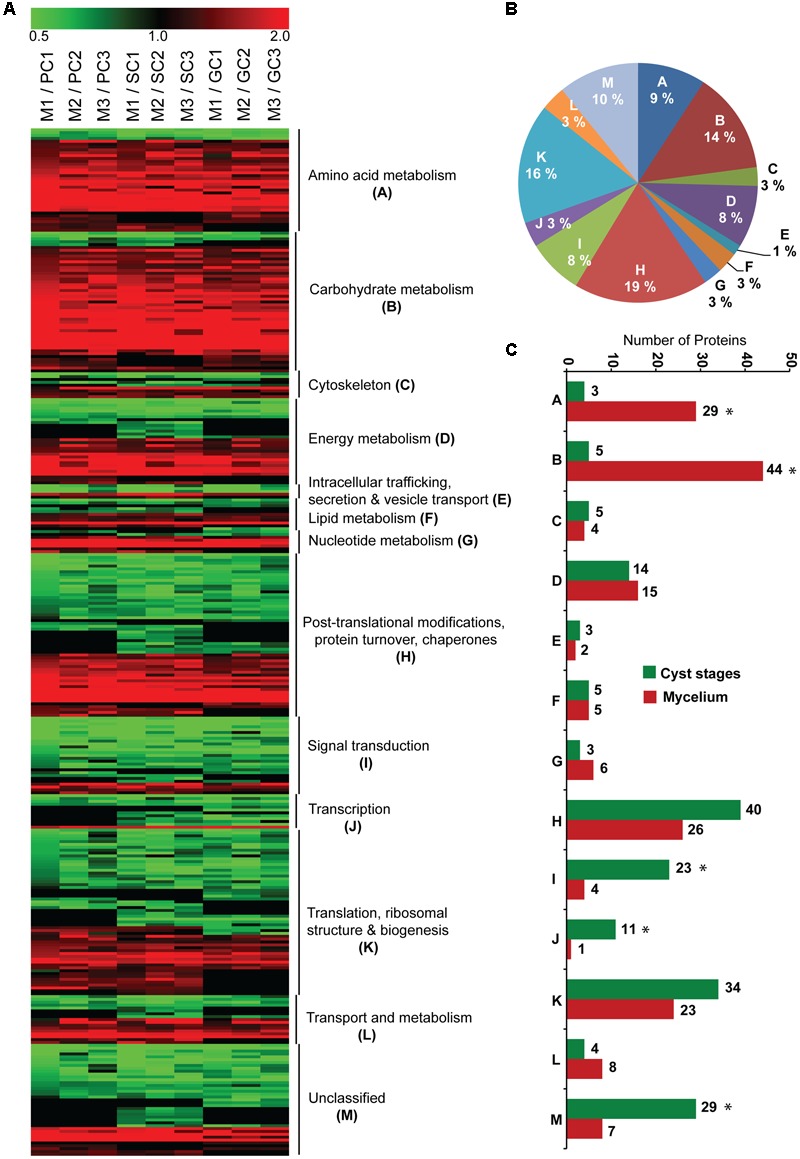
Distribution of functional classification among enriched proteins. **(A)** Heat map illustrating the relative abundance of the significantly enriched proteins in the mycelium (red) and different cyst stages (green) in all three biological replicates. Missing values are shown in black; **(B)** proportional representation of each functional classification among the proteins enriched in the mycelium and different cyst stages of *S. parasitica* combined; **(C)** total number of significantly enriched proteins, associated with each functional category, in the mycelium (red) and different cyst stages (green) of *S. parasitica.* The asterisks mark the functional categories of proteins that present significantly different levels of expression between the mycelium and cyst stages (*p* < 0.05; Chi-square test).

The iTRAQ-based quantitative proteomic analysis revealed significant differences in protein abundance between the four developmental stages of *S. parasitica* (**Figure 3** and Supplementary Table S1). Interestingly, most of the proteins in the PC, SC, and GC samples presented similar enrichment levels, distinguishing the cysts from the mycelium (**Figure 3**). Compared to the mycelium, 110 proteins were enriched in all three cyst samples (PC, SC, and GC). Conversely, compared to the three cyst stages, the mycelium was enriched in 133 proteins (Supplementary Table S1). These data indicate significant quantitative differences between the mycelium and the cysts. However, the different cyst stages were not rigorously identical. Indeed, compared to the mycelium 34 proteins were specifically enriched in two of the three cyst samples and 35 were enriched in only one of the three types of cysts analyzed (Supplementary Table S1). In addition, among the proteins enriched in the mycelium compared to the PC and SC samples, the abundance of three proteins (a ribosomal and two hypothetical proteins) was found to be the highest in the GC samples (Supplementary Table S1).

**FIGURE 4:**
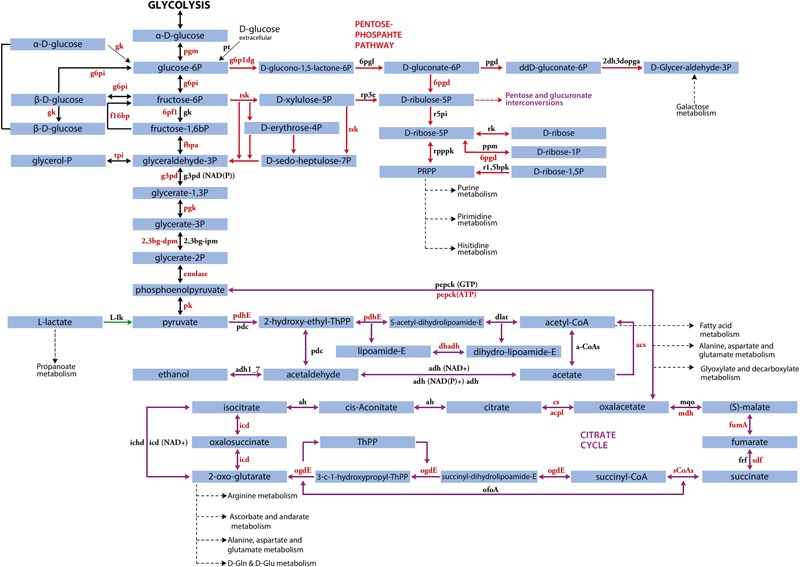
*Saprolegnia parasitica* carbon metabolism. Shown are metabolites and enzymes of the carbon metabolism covering glycolysis, the pentose-phosphate pathway, and citrate cycle. Boxes represent metabolites, arrows indicate enzyme reactions. Enzymes enriched in *S. parasitica* mycelium are colored in red. pgm, phosphoglucomutase; gk, glucokinase; g6pi, glucose-6-phosphate isomerase; f1,6bp, fructose-1,6-bisphosphatase; fbpa, fructose-bisphosphate aldolase; tpi, triosephosphate isomerase; g3pd, glyceraldehyde 3-phosphate dehydrogenase; g3pd(NAD(P)), glyceraldehyde 3-phosphate dehydrogenase (NAD(P)); pgk, phosphoglycerate kinase; 2,3bg-dpm, 2,3-bisphosphoglycerate-dependent phosphoglycerate mutase; 2,3bg-ipm, 2,3-bisphosphoglycerate-independent phosphoglycerate mutase; pepck(GTP), phosphoenolpyruvate carboxykinase (GTP); pepc(ATP), phosphoenolpyruvate carboxykinase (ATP); pk, pyruvate kinase; 6pf1, 6-phosphofructokinase 1; pt, phosphotransferase; L-ld, L-lactate dehydrogenase; pdhE, pyruvate dehydrogenase E1 component; pdc, pyruvate decarboxylase; a-CoAs, acetyl-CoA synthetase (ADP-forming); dlat, pyruvate dehydrogenase E2 component (dihydrolipoamide acetyltransferase); acs, acetyl-CoA synthetase; adh (NAD+), aldehyde dehydrogenase (NAD+); adh (NAD(P)+), aldehyde dehydrogenase (NAD(P)+); adh, aldehyde dehydrogenase; adh1_7, alcohol dehydrogenase 1/7; g6p1dg, glucose-6-phosphate 1-dehydrogenase; 6pgl, 6-phosphogluconolactonase; 6pgd, 6-phosphogluconate dehydrogenase; tsk, transketolase; rp3e, ribulose-phosphate 3-epimerase; r5pi, ribose 5-phosphate isomerase A; rk, ribokinase; rpppk, ribose-phosphate pyrophosphokinase; ppm, phosphopentomutase; r1,5bpk, ribose 1,5-bisphosphokinase; pgd, phosphogluconate dehydratase; 2dh3dopga, 2-dehydro-3-deoxyphosphogluconate aldolase; pfoA, pyruvate ferredoxin oxidoreductase alpha subunit; dhadh, dihydrolipoamide dehydrogenase; ah, aconitate hydratase; cs, citrate synthase; acpl, ATP citrate (pro-S)-lyase; mdh, malate dehydrogenase; mqo, malate dehydrogenase (quinone); fumA, fumarate hydratase, class I; sdf, succinate dehydrogenase (ubiquinone) flavoprotein subunit; frf, fumarate reductase flavoprotein subunit; sCoAs, succinyl-CoA synthetase alpha subunit; odgE, 2-oxoglutarate dehydrogenase E2 component (dihydrolipoamide succinyltransferase); dld, dihydrolipoamide dehydrogenase; ofoA, 2-oxoglutarate ferredoxin oxidoreductase subunit alpha; ogdE, 2-oxoglutarate dehydrogenase E1 component; icd, isocitrate dehydrogenase; icd (NAD+), isocitrate dehydrogenase (NAD+); ichd, isocitrate–homoisocitrate dehydrogenase.

The proteins significantly enriched in the different *S. parasitica* life cycle stages examined were divided into 12 functional categories (**Figure 3**). The largest categories were primarily related to amino acid metabolism (9%), carbohydrate metabolism (14%), energy metabolism (8%), post-translational modifications, protein turnover and chaperones (19%), signal transduction (8%), and translation, ribosomal structure, and biogenesis (16%) (**Figures 3A,B**). However, the enrichment of proteins across functional categories varied between the mycelium and cyst stages (PC, SC, and GC) (**Figures 3A,C**). More specifically, the mycelium contained 29 and 44 enriched proteins associated with amino acid and carbohydrate metabolism, respectively, compared to 4 and 5 proteins only in the cyst samples (**Figure 3C**). The cyst stages were also enriched in proteins that occurred in minute amounts in the mycelium. The most representative examples are proteins involved in post-translational modifications, protein turnover, and chaperones (39 proteins vs. 26 in mycelium); signal transduction (23 vs. 4 in mycelium); transcription (11 vs. 1 in mycelium); and translation, ribosomal structure, and biogenesis (34 vs. 23 in mycelium) (**Figure 3C**). In addition, the mycelium and cysts were respectively enriched in 7 and 29 proteins that could not be classified into any known functional category (**Figure 3C**). KEGG pathway maps are not available for *S. parasitica*. Thus, we used the automatic genome annotation and pathway reconstruction server from the KAAS website^5^ to identify possible enrichment in specific pathways. **Figure 4** illustrates the data obtained for carbon metabolism.

### Quantitative PCR Analysis of Selected Genes in the Mycelium and Secondary Cysts

**FIGURE 5:**
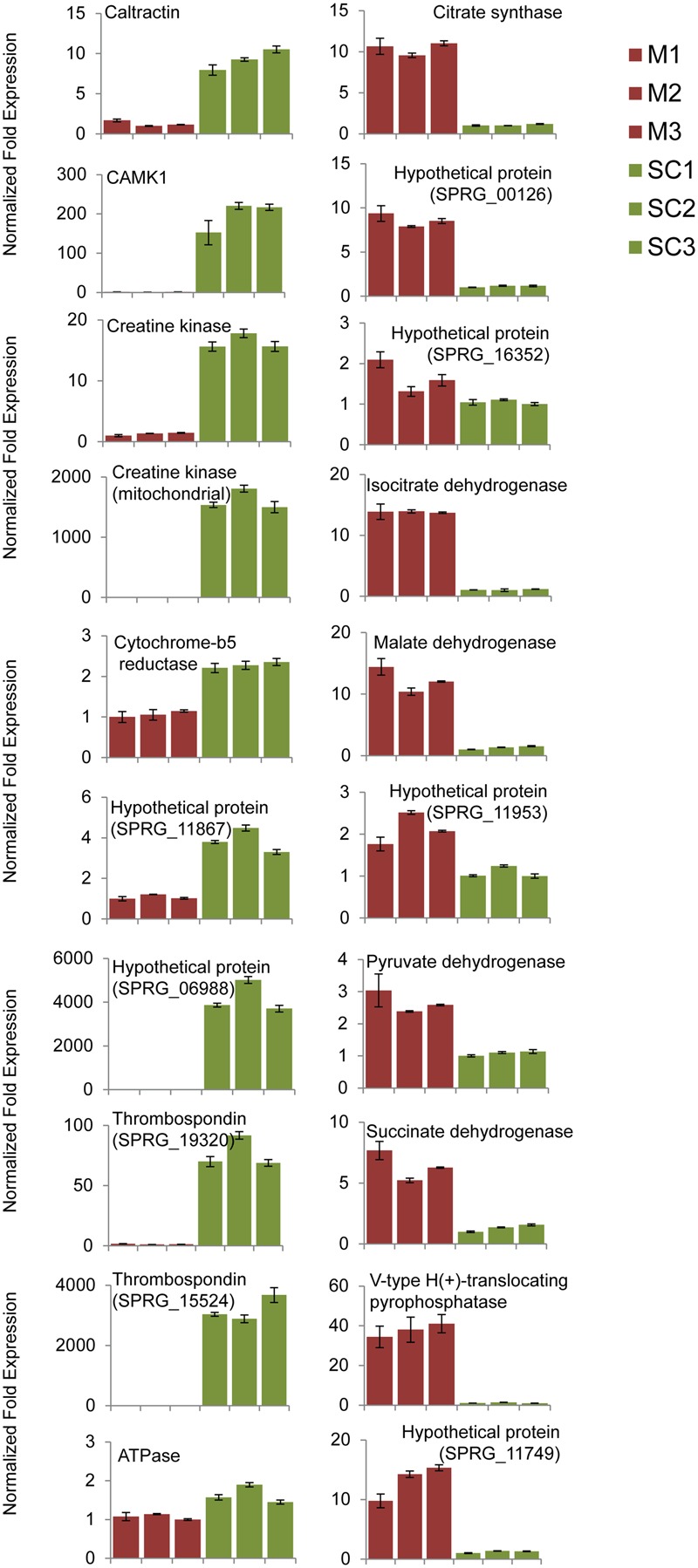
Relative transcript abundance in the mycelium (M) and secondary cysts (SC) of *S. parasitica*. Gene expression was analyzed using quantitative RT-PCR. Relative expression levels were calculated by normalizing the data to the geometric mean of tubulin (SPRG_10729) and elongation factor (SPRG_10439). The vertical bars represent the standard errors of the mean normalized expression value for three separate biological replicates. Description and the corresponding accession number for each gene are shown in Supplementary Table S1.

The relative levels of expression of genes encoding selected proteins enriched in the mycelium or the cysts were compared by qPCR analysis. A total of 20 genes related to carbohydrate metabolism, energy metabolism, and signal transduction were selected for analysis, i.e., 10 corresponding to proteins enriched in the mycelium and 10 to proteins enriched in all three cyst stages (Supplementary Table S1). As the enrichment profiles corresponding to the three cyst stages (PC, SC, and GC) were similar (**Figure 3**), only one cyst sample (SC) was used for the analysis. Their relative expression levels corroborated the iTRAQ data for 18 of the 20 genes examined (**Figure 5** and Supplementary Table S1). Conflicting with the iTRAQ data, the expression level of one ATPase was slightly higher in SC compared to the mycelium (**Figure 5**). Likewise, compared to SC, the mRNA level for the hypothetical protein SPRG_17149 was higher in the mycelium despite iTRAQ experiments supporting a higher abundance in cysts (**Figure 5** and Supplementary Table S1).

## Discussion

Genome analyses revealed that approximately 969 proteins from *S. parasitica* are predicted to be secreted (Tyler et al., 2006; Haas et al., 2009; Jiang et al., 2013). Of these, 131 were identified in this study (Supplementary Table S1) including disintegrins, the ricin B lectin, family 1 carbohydrate binding module (CBM1) and peptidases. Compared to other oomycete species, these protein families are either unique to or enriched in the *S. parasitica* proteome and include some putative pathogenicity factors (Jiang et al., 2013) (Supplementary Table S1). Other secreted proteins/virulence factors identified in this study include glycoside hydrolases, elicitins, peptidases, trypsin, galactose-binding lectins (Gal_lectins), EGF2 and several proteins of unknown function (Supplementary Table S1). The disintegrins, Gal_lectins and other proteins that, among oomycetes, are unique to *S. parasitica*, are more similar to animal pathogenesis-associated proteins (Jiang et al., 2013). It has been hypothesized that these proteins originated in *S. parasitica* via HGT from either the host or other animal pathogens (Jiang et al., 2013). Gal_lectins, for example, are found on fish eggs and skins (Ogawa et al., 2011) and, as they are highly expressed in pre-infection and infection stages, it is conceivable they assist in the adhesion and invasion of fish cells (Jiang et al., 2013). CBM1 and elicitins, also found in *Saprolegnia* species, act as pathogen-associated molecular patterns (PAMPs) in plant pathogenic oomycetes (Gaulin et al., 2006; Lévesque et al., 2010). In *Phytophthora* and *Pythium* species, elicitins appear to be involved in the uptake of sterols from host membranes (Osman et al., 2001) and also induce hypersensitive cell death (Hardham and Blackman, 2010). The elicitins may have other sterol-related functions in *Saprolegnia* pathogenesis (Tyler et al., 2006). Notably, several common effector proteins, e.g., RXLR, CHXC, LFLAK, and Crinklers, known to mediate entry into plant cells and promote infection (Haas et al., 2009; Jiang and Tyler, 2012) are absent in *S. parasitica* (Jiang et al., 2013). In contrast, compared to other eukaryotic pathogens, the *S. parasitica* proteome contains many proteases (Jiang et al., 2013). We identified 77 proteases (peptidases, trypsin, and aspartyl protease) in this study (Supplementary Table S1), which have a potential role in virulence (Butler et al., 2006; Jiang and Tyler, 2012; Jiang et al., 2013). The presence and activity of proteases in the secretome was previously verified by incubating *S. parasitica* culture filtrates with trout immunoglobulin (Ig) M and observing the degradation of the antibody (Jiang et al., 2013).

*Saprolegnia parasitica* is reported to contain one of the largest known kinomes (Jiang et al., 2013). Here, we have identified 37 protein kinases, including Ca^2+^/calmodulin-dependent, tyrosine kinase-like, and CMGC protein kinases (Supplementary Table S1). Twelve of these contained predicted transmembrane domains, suggesting they act as cell surface receptors and play a role in the recognition of extracellular signals (Jiang et al., 2013).

### Proteins Enriched in the Mycelium

Enzymes that catalyze steps of glycolysis, gluconeogenesis, the pentose phosphate pathway, the tricarboxylic acid (TCA) cycle and oxidative phosphorylation were enriched in the mycelium compared to the cysts (PC, SC, and GC) (**Figures 3, 4**). Furthermore, pyruvate dehydrogenase subunits E1 (SPRG_00756) and E2 (dihydrolipoyl transacetylase, SPRG_19552), which link the glycolysis pathway to the TCA cycle, were also more abundant in the mycelium as were enzymes unique to gluconeogenesis, including pyruvate carboxylase (SPRG_00135), phosphoenolpyruvate carboxykinase (SPRG_12635) and fructose bisphosphatase (SPRG_01939) (**Figure 4**). Quantitative PCR analysis further supported the proteomics data, with an increased expression of genes that code for proteins involved in the TCA cycle, namely succinate dehydrogenase (SPRG_14325), citrate synthase (SPRG_13131), isocitrate dehydrogenase (SPRG_01402), and malate dehydrogenase (SPRG_12496) (**Figure 5**). Altogether, these data indicate that the energy required for hyphal growth primarily arises from sugar degradation and interconversion into other metabolites.

Several V-type ATPases were particularly enriched in the mycelium compared to the cysts (Supplementary Table S1). In animal and yeast cells, these ATP-driven proton pumps acidify organelles, which is critical for the function of the secretory and endocytic pathways (Kane, 2006; Huang and Chang, 2011). Similarly, in *Arabidopsis thaliana*, V-ATPase activity in the *trans*-Golgi network/early endosome is required for exocytosis and recycling (Luo et al., 2015). Although no oomycete V-ATPases have been biochemically characterized to date, the fact that these proteins are highly conserved across kingdoms suggests that they perform similar functions in *S. parasitica* as in their animal, yeast, and plant counterparts.

The higher abundance of pyrophosphatase (PPase; SPRG_05568 and SPRG_00126) in the mycelium, supported by higher transcript levels (**Figure 5**), suggests an important role of this enzyme in hyphal growth. This is supported by the observation that accumulation of PPi due to defects in PPase activity leads to reduced cell growth (Chen et al., 1990; Lundin et al., 1991; Ko et al., 2007; Martinoia et al., 2007; Bertoni, 2011; Ferjani et al., 2011).

Family C1, M13, 16-18, and S8-10 peptidases, proteasome alpha/beta subunits, and redox enzymes such as peroxiredoxin, glutaredoxin, and glutathione *S-*transferase were enriched in the mycelium (Supplementary Table S1). Genes encoding peptidases are highly expressed in the mycelium of *S. parasitica* and the corresponding peptidases are able to degrade trout IgM (Jiang et al., 2013). It has been suggested that oomycete peptidases contribute to virulence by avoiding pathogen recognition by the fish immune system, thereby promoting successful mycelial colonization of the host tissues (Banfield and Kamoun, 2013). Alternatively, enrichment of peptidases in the mycelium may reflect a role in nutrition in environments rich in protein.

### Proteins Enriched in the Cysts

Proteins that are clearly enriched in all three cyst samples compared to the mycelium are involved in signal transduction and transcription (**Figure 3C**). These include protein kinases, disintegrins, thrombospondins, WD40-repeat containing proteins, and calcium-binding proteins such as EGF-domain-containing proteins, and EF-hand-containing proteins (Supplementary Table S1).

As mentioned earlier, *S. parasitica* has a very large kinome and approximately 10% of kinase-encoding genes are induced upon infection of a host (Jiang et al., 2013). However, our proteomic data show that only two protein kinases were significantly enriched in the cyst samples compared to the mycelium (Supplementary Table S1). The increased expression of the Ca^2+^/calmodulin-dependent protein kinase gene (SPRG_10463) in secondary cysts was also confirmed by q-PCR (**Figure 5**).

Within oomycetes, disintegrins appear to be unique to *S. parasitica* (Jiang et al., 2013). They are highly expressed in pre-infectious stages of the pathogen and may play an important role in its interaction with the host (Jiang et al., 2013). This hypothesis is further supported by our data which show that, out of the 16 disintegrins predicted from the genome of the pathogen, 4 are enriched in the pre-infectious cyst stages (Supplementary Table S1).

Compared to the mycelium, both transcript and protein levels corresponding to accessions SPRG_15524 and SPRG_19320 were much higher in all cyst samples (**Figure 5** and Supplementary Table S1). Sequence analysis revealed that both proteins are predicted to contain the 50-amino-acid thrombospondin type-1 repeat (TSR1), which occurs in adhesive proteins secreted by mammalian cells and malarial parasites (Tomley and Soldati, 2001; Robold and Hardham, 2005). In both animals and plants, the establishment of an infectious disease involves adhesion of the pathogen cells to the host surface (Klein, 2000; Tucker and Talbot, 2001). Thrombospondin motifs are typically found in proteins present in the extracellular matrix, where intercellular adhesion occurs (Adams and Tucker, 2000). Based on these observations and the expression patterns of SPRG_15524 and SPRG_19320 in cysts (**Figure 5** and Supplementary Table S1), it can be hypothesized that TSR1-containing thrombospondin from *S. parasitica* play a role in adhesion to fish cells during the initial phase of the infection process.

Flagella play important roles in motility, sensory perception and life cycle control in eukaryotes, including protists (Ginger et al., 2008). It is also known that the flagellar proteome is rich in motor and signal transduction components (Pazour et al., 2005). Interestingly, our study shows that the flagellar protein caltractin (SPRG_09593), two other Ca^2+^-binding proteins (SPRG_09020 and SPRG_07497), one intra-flagellar transport protein (SPRG_04104) and tubulin (SPRG_09503) are enriched in the cysts (Supplementary Table S1). Altogether these observations are consistent with a role of these proteins in the movement of zoospores, which originate from primary and secondary cysts (van West, 2006).

Additional proteins specifically enriched in the cysts are associated to ‘energy metabolism’ (**Figure 3C** and Supplementary Table S1). These include three creatine kinases which reversibly transfer a phosphate group from phosphocreatine to ADP to produce ATP (Wallimann et al., 1992; Wallimann and Hemmer, 1994). Q-PCR analysis confirmed the quantitative proteomics data, with one mitochondrial creatine kinase (SPRG_02985) exhibiting a 1000-fold higher expression in the secondary cysts compared to the mycelium (**Figure 5**). In animals, the creatine kinase/phosphocreatine system plays a key role in muscles by controlling energy homeostasis (Wallimann et al., 1992). Interestingly, compared to hyphal cells, creatine kinases are highly expressed in the sporangia of *P. infestans* (Kim and Judelson, 2003) and in germinating cysts from both *Phytophthora pisi* and *Phytophthora sojae* (Hosseini et al., 2015). These observations suggest that the cyst stages of oomycetes require rapid energy production for their cellular metabolism and that the creatine kinase system of these microorganisms, including *S. parasitica*, may fulfill this function by further increasing the amount of cellular ATP.

Protein synthesis and cytoskeleton formation are important processes for cyst germination and early infection in *Phytophthora* spp. (Ebstrup et al., 2005; Savidor et al., 2008). Consistent with this observation, our data show that many ribosomal proteins and related proteins such as the eukaryotic initiation factor, RNA processing factor 31, RNA recognition motif-containing proteins, and cytoskeletal proteins are enriched in the *S. parasitica* cysts (Supplementary Table S1). Several related proteins involved in protein folding and belonging to the category ‘post-translational modifications, protein turnover, and chaperones’ were also more abundant in the cyst stages. These include heat shock proteins, calreticulin, protein disulfide isomerase, peptidyl prolyl *cis-trans*-isomerase, tetratricopeptide repeat containing protein, chaperonin and prefoldins (Supplementary Table S1).

It should also be noted that the actual protein profile associated with Saprolegnia infection can be different during its pathogenic development on fish, compared to this study where protein profiling was performed during cyst development and germination *in vitro*. Nevertheless, as the PC, SC, and GC are the pre-infectious stages of *S. parasitica*, some of the proteins enriched in these cells represent potential targets for the development of new strategies for disease control.

Several significantly enriched proteins in the mycelium and cysts are annotated as ‘hypothetical’ and contain domains of unknown functions. Some of these proteins may play a critical role in specific life cycle stages of *S. parasitica* and should be further characterized using targeted approaches.

## Author Contributions

VS and VB designed the research. SR and VS performed the research and analyzed the data. VS and VB wrote the manuscript with inputs from SR.

## Conflict of Interest Statement

The authors declare that the research was conducted in the absence of any commercial or financial relationships that could be construed as a potential conflict of interest.

## Footnotes

1 http://weizhong-lab.ucsd.edu/metagenomic-analysis/server/

2 http://www.genome.jp/kegg/kaas/

3 http://www.ncbi.nlm.nih.gov/Structure/cdd/wrpsb.cgi

4 http://weizhong-lab.ucsd.edu/metagenomic-analysis/server/kog/

5 http://www.genome.jp/kegg/kaas/
