# Supplementary material for: Quantitative Proteomic Analysis of Four Developmental Stages of Saprolegnia parasitica
Source: Front Microbiol. 2018 Jan 11;8:2658. doi: 10.3389/fmicb.2017.02658 (PMC5768655; doi:10.3389/fmicb.2017.02658)
Supplement: Supplementary file 2 [file Data_Sheet_1.PDF]

Quantitative proteomic analysis of four developmental stages of *Saprolegnia parasitica*

Vaibhav Srivastava, Svetlana Rezinciuc & Vincent Bulone

Supplementary Figures

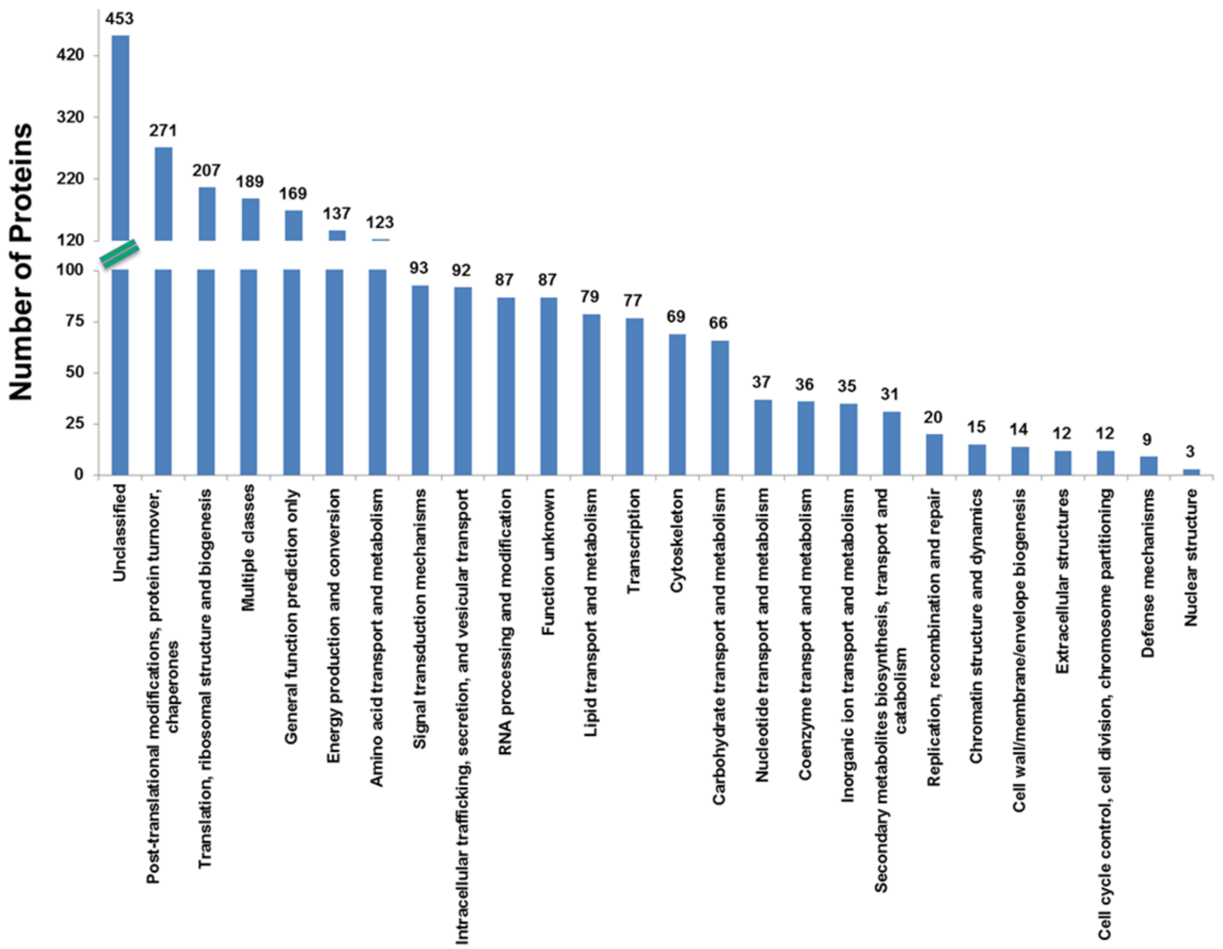

Supplementary Figure S1: Functional classification of all 2423 identified proteins in *Saprolegnia parasitica*. The KOG (EuKaryotic Orthologous Groups) database was used for functional classification of all proteins.

Quantitative proteomic analysis of four developmental stages of *Saprolegnia parasitica*

Vaibhav Srivastava, Svetlana Rezinciuc & Vincent Bulone

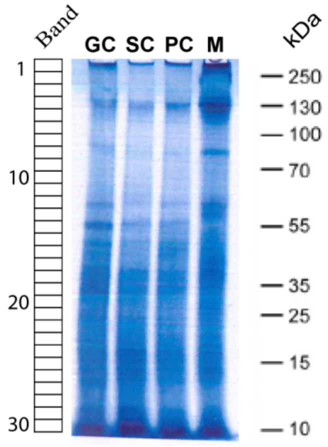

Supplementary Figure S2: SDS-polyacrylamide gel electrophoresis analysis of the proteins extracted from the mycelium (M), germinated cysts (GC), secondary cysts (SC) and primary cysts (PC) of *Saprolegnia parasitica*. Each lane of the Coomassie-blue-stained gel was cut into 30 bands as shown on the left side of the picture.

Quantitative proteomic analysis of four developmental stages of *Saprolegnia parasitica*

Vaibhav Srivastava, Svetlana Rezinciuc & Vincent Bulone

Legends of Supplementary Table S1:

Results are shown in the following sheets:

1. *Significantly-Enriched-Proteins:* List of *Saprolegnia parasitica* proteins significantly enriched in the mycelium and other cyst stages in all three biological replicates (from iTRAQ-experiments).

2. *BR1-iTRAQ-peptides:* List of all unique proteins together with their corresponding peptides, identified by iTRAQ experiments from the first biological replicate (BR1) of Mycelium and Cyst (primary cyst, secondary cyst and germinated cyst) samples.

3. *BR1-iTRAQquant*: List of all unique proteins quantified by iTRAQ experiments from the first biological replicate (BR1) of Mycelium and Cyst (primary cyst, secondary cyst and germinated cyst) samples.

4. *BR2-iTRAQ-peptides:* List of all unique proteins together with their corresponding peptides, identified by iTRAQ experiments from the second biological replicate (BR2) of Mycelium and Cyst (primary cyst, secondary cyst and germinated cyst) samples.

5. *BR2-iTRAQquant*: List of all unique proteins quantified by iTRAQ experiments from the second biological replicate (BR2) of Mycelium and Cyst (primary cyst, secondary cyst and germinated cyst) samples.

6. *BR3-iTRAQ-peptides:* List of all unique proteins together with their corresponding peptides, identified by iTRAQ experiments from the third biological replicate (BR3) of Mycelium and Cyst (primary cyst, secondary cyst and germinated cyst) samples.

7. *BR3-iTRAQquant:* List of all unique proteins quantified by iTRAQ experiments from the third biological replicate (BR3) of Mycelium and Cyst (primary cyst, secondary cyst and germinated cyst) samples.

8. *Total-Proteins:* Combined list of all Saprolegnia unique proteins obtained from in-gel and solution (iTRAQ experiments) proteomics analysis.

9. *Q-PCR primers*: List of oligonucleotide primer pairs used in real-time RT-PCR.
